# Insights and future directions: Applying the One Health approach in international agricultural research for development to address food systems challenges

**DOI:** 10.1016/j.onehlt.2025.101007

**Published:** 2025-02-27

**Authors:** Hung Nguyen-Viet, Steven Lâm, Silvia Alonso, Fred Unger, Arshnee Moodley, Bernard Bett, Eric M. Fèvre, Theodore Knight-Jones, Siobhan M. Mor, Ha Thi Thanh Nguyen, Delia Grace

**Affiliations:** a International Livestock Research Institute, Nairobi, Kenya; b International Livestock Research Institute, Addis Ababa, Ethiopia; c International Livestock Research Institute, Hanoi, Viet Nam; d Department of Veterinary and Animal Sciences, University of Copenhagen, Frederiksberg C, Denmark; e Institute of Infection, Veterinary and Ecological Sciences, University of Liverpool, Neston, United Kingdom; f Department of Medical Biochemistry and Microbiology, Uppsala University, Uppsala, Sweden; g Natural Resources Institute, University of Greenwich, Greenwich, United Kingdom

**Keywords:** One health, Food systems, Antimicrobial resistance, Zoonoses, Food safety, Evaluation

## Abstract

For more than 15 years, the International Livestock Research Institute (ILRI) has been striving to understand and address One Health challenges at the intersection of livestock, humans, and the environment. We present an overview of ILRI One Health projects implemented with partners across Asia and Africa, reflecting on key learnings and future directions for One Health research and food systems transformation. Drawing on a review of peer-reviewed and grey literature, we analyzed processes and outcomes of ILRI-led and supported initiatives using a realist evaluation framework (context, mechanisms, outcomes), and present insights within select One Health topic areas such as zoonoses, food safety, antimicrobial resistance. Our findings emphasize the need for stronger cross-sectoral collaboration, greater engagement with policymakers to translate research findings into actionable strategies, and the development of adaptable and context-specific interventions.

## 1 Background

CGIAR (former name of *Consultative Group on International Agricultural Research*) is a global research partnership dedicated to transforming food, land, and water systems in a climate crisis. Among the 15 centers, the International Livestock Research Institute (ILRI) operates in low- and middle-income countries (LMICs) with the core mandate of supporting poor farming communities to produce healthy livestock to generate resources to improve livelihoods, reduce poverty and provide more nutritious high-quality protein and micronutrients for the community. One of the biggest challenges to the health and livelihood services which livestock systems offer are zoonotic diseases that spread between animals and humans (https://www.ilri.org/).

A One Health approach offers a promising framework for tackling health threats stemming from the interface between animals, humans, and the environment [1]. Acknowledging the interconnectedness of these elements, One Health calls for collaboration across sectors and disciplines [2]. As the only CGIAR center exclusively dedicated to livestock systems and their interactions with wildlife and the environment, ILRI has been leading research to address One Health challenges within agricultural settings across the CGIAR.

**Fig. 1:**
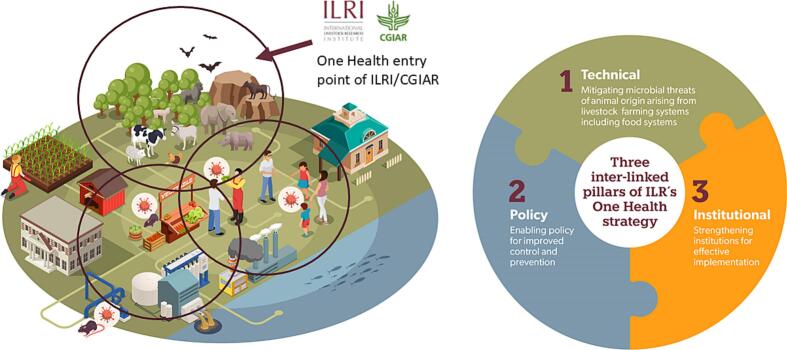
ILRI One Health framework and three pillars of the strategy leading integrated science-innovation-policy driven One Health approaches to address food system challenges [3].

ILRI brings 50 years of experience in zoonoses, animal health, and food systems. Headquartered in Nairobi, Kenya, and co-hosted by the Government of Ethiopia, ILRI is equipped with state-of-the-art laboratories and a comprehensive research infrastructure, including a specialized center focused on environmental issues [3]. This positions ILRI at the forefront of the One Health triad, representing two key sectors: animals and the environment (Fig. 1). Additionally, ILRI benefits from a vast network of partnerships, collaborating with public health institutions in Africa, Asia, and around the world. Although ILRI did not always explicitly adopt a One Health approach, it has been working to address One Health issues for many years – particularly zoonoses and food safety, and more recently antimicrobial resistance (AMR).

Here, we present an overview of One Health initiatives implemented with ILRI partners across Asia and Africa, drawing on a review of the published and grey literature on these initiatives, as well as on our own experiences at ILRI working within One Health. Our objective is to share lessons learned and future directions from applying the approach in agricultural settings. We hope this paper may improve the success of future efforts wanting to use a One Health approach to address interconnected challenges in health and create healthier food systems.

## 2 Methods

We examined the published and grey literature to provide insights into One Health initiatives led or supported by ILRI. We searched for documents using CGSpace, a repository of CGIAR and partner research outputs and knowledge products. We used the following search string “One Health” with no restrictions place (e.g. on date, location) until October 2024. We also drew on unpublished reports which came from the authorship team, many of whom worked on ILRI One Health initiatives. Initiatives were considered relevant if they: described the process or outcomes of the initiative; were a relatively large project (we defined as having received at least 1 million USD in funding); used explicitly a One Health approach; and, were reported on in a journal article or report.

We captured general characteristics of the initiative including goal, country, focal area, funding, and duration. An important tool in international development and agricultural research programs is theory-driven evaluation, which aims to develop a “program theory” of how a program works and how the various components interact to produce outcomes [4]. For example, realist evaluation encourages the exploration of mechanisms that are likely to operate in social programs, contexts in which they might operate, and outcomes that will be observed if they operate as expected [5]. Using realist evaluation as a framework, we extracted information on initiative context, mechanisms, and outcomes. We synthesized and presented findings broadly along these domains, and linked the findings back to ILRI strategies, processes, and practices.

### 2.1 Context: history of One Health work at ILRI

ILRI first advocated for a One Health approach in 2006, when colleagues emphasized the importance of including the often overlooked zoonotic diseases as part of global health programs [6,7]. ILRI worked to ensure projects identified or designed control methods for zoonotic disease that were accessible and more relevant to low-resource settings. Although not a large-scale project, ILRI also held an “African Challenge Dialogue” to highlight the need to link climate and health research towards identifying solutions to fight infectious diseases – including zoonoses – in Africa. This dialogue recommended a One Health approach [8].

ILRI first adopted an EcoHealth approach in 2008 with the launch of the Ecosystem approach to better management of zoonotic diseases (EcoZD) project (2008–2014), a framework closely aligned with One Health principles [9]. The CGIAR Research Program (CRP) on Agriculture for Nutrition and Human Health (A4NH), initiated in 2012, further accelerated the integration of an interdisciplinary, One Health approach within CGIAR [10]. ILRI's core research areas have traditionally included zoonoses, food safety, and AMR, considered “classic” One Health issues. ILRI One Health strategy was published in 2022 to position One Health work in CGIAR and globally to address health challenges at the interface of people, animal and the environment [3].  Below, we discuss how the One Health approach has served as a framework for addressing these challenges within ILRI research and provide a summary of ILRI initiatives (see Table 1 and Fig. 1).

**Table 1:** Overview of One Health initiatives led or supported by ILRI.

Initiative	Objective	Countries	Focal area(s)	Funding; duration
Cross-cutting One Health initiatives				
Co-infection with Rift Valley fever virus, *Brucella* spp. and *Coxiella burnetii* in humans and animals in Kenya: Disease burden and ecological factors [13]	To analyse existing samples and carry out active surveillance to determine the burden of zoonoses in humans, livestock and wildlife in Kenya	Kenya	Three key areas: • Rift Valley fever • Brucellosis • Q fever	2.1 M USD; September 2019 to December 2023
Capacitating One Health in Eastern and Southern Africa (COHESA) [14]	To generate society-wide capacity to deliver solutions to One Health issues	12 countries in Eastern and Southern Africa: • Botswana • Ethiopia • Kenya • Malawi • Mozambique • Namibia • Rwanda • Somalia • Tanzania • Uganda • Zambia • Zimbabwe	Four work packages: • Knowledge sharing • Governance, • Education and research • Delivery of One Health solutions	9.2 M euros; December 2021 to November 2025
CGIAR Initiative on One Health (OHI) [15]	To show how One Health principles and tools integrated into food systems can benefit health	Eight countries: • Bangladesh • Cote d'Ivoire • Ethiopia • India • Kenya • Uganda • Vietnam • Malawi	Five work packages: • Zoonoses • Food safety • Antimicrobial resistance • Environment (water) • Economics, governance, and behaviour	16.4 M USD; January 2022 to December 2024
One Health regional network for the Horn of Africa (HORN) [16]	To improve the health and wealth of the people of the Horn of Africa	Four countries in the Horn of Africa: • Ethiopia • Eritrea • Kenya • Somalia	Five step process: • Research capability assessments of partner institutions • Training to non-research staff from these institutions • Capacity building of researchers • Conduct basic and applied research • Create One Health Regional network	7.7 M pounds; October 2017 to March 2022
One Health Research, Education and Outreach Centre in Africa (OHRECA) [17]	To enhance health through applied One Health research, capacity building, One Health networks support, and developed pathways from evidence to policy and practice	Seven countries in Sub-Saharan Africa: • Burkina Faso • Ethiopia • Kenya • Malawi • Mali • Senegal • Tanzania	Four themes: • Preventing emerging infectious diseases • Controlling neglected zoonoses • Ensuring safe food • Reducing antimicrobial resistance	15 M euros; January 2020 to December 2025
One Health for Humans, Environment, Animals and Livelihoods (OH4HEAL) [18]	To enhance the well-being of vulnerable communities in (agro)pastoralist areas	Three countries: • Ethiopia • Kenya • Somalia	Three key objectives: • Engage pastoralists in defining One Health Units • Implement One Health Units • Provide evidence on One Health Units as solution for service delivery in pastoralist areas	8 M Swiss Franc; March 2020 to October 2024
Boosting Uganda's Investments in Livestock Development (BUILD) [19]	To support existing livestock health initiatives by helping to scale solutions through research, extension, and partnerships	Uganda	Four themes: • Peste des petits ruminants • Rift Valley fever • Antimicrobial resistance • Veterinary public health at the point of slaughter	7 M euros; January 2019 to December 2023
The CGIAR Research Program on Agriculture for Nutrition and Human Health (A4NH)	To leverage agriculture to improve health and nutrition outcomes	Five focus countries: • Bangladesh • Ethiopia • India • Vietnam • Nigeria	Three key areas: • Consumption of healthy, affordable, and safe foods • Gender and equity • Impact assessment and evaluation • Antimicrobial Resistance • Zoonosis	Phase I (ca. 18 M USD; 2011–16) and phase 2 (14 M USD; 2017–21)

Zoonoses				
Zoonoses in Livestock in Kenya (ZooLinK) [20]	To enable Kenya to develop an effective surveillance programme for zoonoses	Kenya	Several themes: • Zoonoses • Emerging disease • Surveillance • One Health • Policy • Capacity building	3.3 M pounds; January 2017 to December 2021
People, Animals and their Zoonoses (PAZ) [21]	To understand zoonotic and other disease burdens, the distribution of infection, and determinants of infection in both people and their livestock	Two key areas: • Kenya • Countries in Europe	Several themes: • Zoonoses • Co-infection • One Health • Animals, livestock, wildlife • Capacity building	1 M USD; January 2009 to December 2012
Ecosystem approaches to the better management of zoonotic emerging infectious diseases in the Southeast Asia region (EcoZD) [22]	To increase the capacity of researchers and implementers to use novel ecohealth approach for better control of zoonoses	Six countries in Southeast and East Asia: • Cambodia • China • Indonesia • Laos • Thailand • Vietnam	Several themes: • Improving understanding of zoonotic diseases • Promoting the EcoHealth approach • Capacity building to respond to zoonotic diseases • Informing policies and actions	5 M CAD; February 2008 to July 2013
Epidemiology, ecology and socio-economics of disease emergence in Nairobi (Urban Zoo) [23]	To understand the mechanisms that may lead to the introduction of pathogens into urban populations and their subsequent spread	Kenya	Three main themes: • Livestock as sources of pathogens • Landscape genetics approach • Public health	2.5 M pounds; February 2012 to January 2017
Improving human health through sustainable value chains using ICT in Vietnam (ICT4Health) [24]	To tackle zoonotic diseases by strengthening capacities of national partners in surveillance, preparedness, and disease prevention and control	Vietnam	Five key activities: • Prioritization of zoonoses • Establishment of surveillance working groups • Pilot surveillance systems • Develop tools to improve surveillance • Implement community-based interventions	3 M USD; January 2022 to December 2026

Food safety				
Market-based approaches to improving the safety of pork in Vietnam (SafePORK) [25]	To reduce the burden of food-borne disease in informal, emerging and niche markets	Vietnam	Four key activities: • Generate evidence on health and economic burden of food-borne diseases • Build a theory of change for how food safety can be achieved • Pilot light-touch interventions, taking into account gender equity • Build capacity in risk management	2 M AUD; October 2017 to June 2023
Pull-Push: Urban food markets in Africa – Incentivising food safety using a Pull-Push approach [26]	To assess if consumer demand for food safety can be generated and harnessed to drive improvements in food safety in African informal urban food systems	Two countries: • Burkina Faso • Ethiopia	Several themes: • Understand and assess microbial food safety risks and burdens in chicken and vegetable value chains • Deliver consumer food safety awareness campaigns and assess their impact of consumer food safety practices • Train regulators in food safety • Develop and assess value chain interventions to improve food safety	4.7 M USD; October 2018 to November 2023
Making the most of milk (MoreMilk) [27]	To improve child health and nutrition outcomes through milk consumption	Two countries in East Africa: • Kenya • Tanzania	Two key activities: • Train milk vendors on milk safety as well as business skills and marketing • Monitor milk quality, vendor business performance, and nutrition	4.6 M; October 2016 to September 2023
Reducing disease risks and improving food safety in smallholder pig value chains in Vietnam (PigRISK) [28]	To improve the livelihoods of smallholder pig farmers through improving market access and food safety in the pork value chain	Vietnam	Three key objectives: • Evidence on health risks from pork value chain • Develop and test incentive-based innovations • Increase capacity to manage risks	1.9 M AUD; June 2017 to September 2017
Estimating the burden of foodborne disease in Ethiopia (TARTARE) [29]	To reduce morbidity and mortality from FBD by developing a risk-based framework.	Ethiopia	Three key objectives: • Understand public health burden and costs of selected pathogens • Search for strategies to reduce risks • Assess where national resources should be allocated	3.4 M USD; January 2022 to December 2023
Vendor Business School [30]	To enhance the business and food handling skills of street vendors.	Two countries: • Kenya • Philippines	Two key objectives: • Training of milk and vegetable vendors, primarily women • Professionalizing of informal food workers	1 M USD; January 2022 to December 2024

Antimicrobial resistance (AMR)				
Fleming Fund Fellowships [31]	To enhance the skills and expertise of individuals (government employees) working in the human, animal, and aquatic health sectors	Three countries: • Zambia • Ghana • Bangladesh	Training and mentorship in AMR diagnostics, AMR and AMU surveillance, and AMR governance	1.67 M USD; January 2020 to February 2023
Fleming Fund AMROH [31]	To provide technical assistance within animal and environmental health to the Country Grants	Eight countries: • Kenya • Uganda • Malawi • Zimbabwe • Eswatini • Rwanda • Tanzania • Zambia	Three key objectives: Provide technical assistance to country grantee in the areas of animal health and environmental surveillance	2.3 M USD; March 2024 to December 2025)
Fleming Fund Country Grant Phase 1 [32]	To enhance AMR and antimicrobial use (AMU) surveillance systems	Kenya	• Strengthen AMR governance • Improve laboratory capacity for antimicrobial susceptibility testing • Strengthen AMR surveillance system in the human and animal health sectors	Phase 1 (7.84 M USD; September 2019 to February 2023); Phase 2 (4 M pounds; March 2024 to December 2025)
Selecting Efficient Farm-level Antimicrobial Stewardship Interventions (SEFASI) [33]	Identifying efficient farm-level interventions to reduce AMU and AMR transmission in livestock	Three countries: • Senegal • Denmark • United Kingdom	Cost-benefit of farm-level interventions to reduce AMU	1.12 M USD, January 2022 to December 2024
R'OHOKET [34]	Raising awareness and promoting AMR prevention actions at local, national, and regional levels	Four countries: • Kenya • Uganda • Ethiopia • Tanzania	Three key objectives: • Promotion of research and evidence-based AMR • Understanding, awareness and prevention actions at local national and regional levels • Strengthening decision-making processes	1.1 M USD, January 2024 to December 2026

#### 2.1.1 Zoonoses

Zoonoses encompass a range of diseases, some of which are considered neglected or endemic, while others are classified as new or emerging. Neglected zoonoses, largely controlled or eliminated in high-income countries (HICs), continue to pose a significant burden on LMICs, whereas emerging zoonoses represent a global threat. ILRI began its zoonoses research in the 1990s, concentrating on neglected zoonoses such as bovine tuberculosis, brucellosis, human African trypanosomiasis (sleeping sickness), and rabies.

In the 2000s, as global concern increased following the pandemic threat from highly pathogenic avian influenza A (H5N1), and the prior emergence of bovine spongiform encephalopathy (mad cow disease) and severe acute respiratory syndrome, ILRI expanded its focus to emerging zoonoses. Research on these diseases has included avian influenza, Ebola virus disease, Middle Eastern respiratory syndrome coronavirus, Rift Valley fever, as well as livestock-related AMR.

As the program evolved, ILRI's zoonoses research transitioned from a predominately veterinary public health paradigm to a One Health approach, emphasizing the interconnectedness of human, animal, and ecosystem health. In addition to focusing on high-priority zoonotic diseases, ILRI's research initiatives also adopted a systems lens to target specific livestock systems, particularly pastoral, smallholder, and urban settings. Many zoonoses, both neglected and emerging, are also foodborne diseases (FBDs), which are discussed in the next section.

#### 2.1.2 Food safety

Until the 2000s, food safety was historically a minor part of ILRI research. This was partly due to a lack of recognition that FBD was a major public health and development issue in LMIC. Donor investments in food safety were small compared with the scale of the problem, with investments in comparable diseases, and with the potential return on investments [11]. Most investments had focused on trade rather than on ensuring the health of consumers in LMICs.

In the 2000s, ILRI began a program on improving human health through livestock research in three areas: (i) animal-source foods for nutrition, (ii) zoonoses, and (iii) FBD. The latter was the first group within CGIAR with an explicit food safety mandate (rather than focusing on specific hazards) and with expertise in using research methods for food safety rather than diseases in general. ILRI was also one of the first groups to focus on food safety in the ‘informal markets’ of LMICs, and by the 2010s, had become the lead research institute globally in this emerging area. Specifically, ILRI developed and contributed to tools, methods, and metrics, including participatory risk assessments and food safety system performance assessments. Technology development and testing was a growing area with a focus on appropriate technologies such as disinfectants and pest control.

From the mid-2010s, ILRI explicitly adopted One Health principles to address food safety issues through testing interventions along livestock value chains, working across veterinary and public health sectors, and exploring the interactions between food safety and other development issues [12].

#### 2.1.3 AMR

AMR is a natural phenomenon present across various environments, but its emergence, selection, and spread are primarily driven by the misuse and overuse of antimicrobials in both animal and human health sectors. AMR poses significant risks to public health, livestock productivity, and environmental health. In humans, it leads to higher mortality rates, prolonged illnesses, and increased healthcare costs. In animals, AMR contributes to reduced productivity and welfare issues, severely affecting livelihoods and food security, particularly in LMICs. Additionally, AMR impacts the environment, with potential links to climate change.

ILRI initially recognized AMR as both a barrier to livestock productivity and a public health concern. Since 2012, ILRI has prioritized ensuring that smallholder farmers have access to effective disease control measures to protect their livestock. Through programs such as the CRP on A4NH, ILRI has focused on understanding antimicrobial use in livestock, its impact on productivity, and the drivers behind AMR in livestock systems. Moreover, ILRI's work within these CRPs and in subsequent projects, employed a One Health approach to explore the transmission of AMR between animal and human health systems, while promoting rational and efficient antimicrobial use that benefits both animal herds and human health. Our research findings highlight the urgent need for sustainable, cross-sectoral interventions that address AMR, safeguard livelihoods, and mitigates the broader environmental impacts, including those related to climate change.

### 2.2 Mechanisms: ILRI's approach to working in One Health

ILRI One Health's goal of improving food security and reducing poverty is achieved through three main, interconnected pillars: technical, policy and institutional [3].

The technical pillar addresses high impact pathogen threats (viruses, bacteria, fungi and parasites) of animal origin under four specific overlapping technical themes, namely: emerging and re-emerging zoonoses; endemic zoonoses; foodborne diseases; and AMR. Technical approaches involve strategic research using a systems approach targeting priority livestock production and food systems, including their interfaces with humans and ecosystems in LMICs. The priority livestock systems targeted are small- to medium-sized mixed livestock/crop and pastoralist systems, their associated livestock value chains, animal markets and formal and informal slaughterhouses and food processing facilities. The policy pillar uses evidence generated from technical research and analysis of social, cultural, gender and economic factors to drive One Health policy for early warning and response and improved prevention and control for mitigating pathogen threats. The institutional pillar promotes the strengthening of health capacities, particularly the animal and ecosystem health sectors that are considered the weakest link in the One Health triangle. This pillar also promotes the strengthening of governance of government and regional institutions to support collective One Health action [3].

ILRI deploys a multi-disciplinary One Health research approach at all levels from planning to implementation. This approach leverages in-house expertise and experience in animal and environment health and draws on diverse disciplines such as social science, economics, anthropology, epidemiology, ecology, and modeling. To ensure robust research outcomes, ILRI forms strategic partnerships with public health sectors at national, regional, and international levels. These collaborations ensure broad sectoral representation in research efforts, particularly in addressing the complex challenges of zoonotic disease, food safety, and AMR prevention and control. ILRI strengthens its extensive network of existing partnerships and establishes new alliances when necessary to support the research and delivery of its outputs. At the country level, ILRI partners with ministries of agriculture, health, environment, commerce, education, and meteorological departments. Engagement with private sector stakeholders, such as farmers, livestock traders, and market owners, supports a farm-to-fork food systems approach. At the international level, ILRI collaborates with key sectoral partners, including the Quadripartite (FAO, WHO, UNEP, WOAH), as well as One Health research centers, platforms, and networks that share similar objectives, reinforcing the global effort to address One Health challenges [35].

The key outputs of this research include the development of surveillance and early warning systems, innovative diagnostic and characterization tools for priority pathogens, and the creation of new vaccines and tailored prevention measures for targeted systems. Additionally, a suite of scientific tools, standards, guidelines, and risk communication and advocacy materials will empower government health services and farming communities to strengthen the resilience of their health systems against diseases. These outputs aim to enhance preparedness and response, improving overall health system sustainability and disease prevention capabilities [36,37].

### 2.3 Outcomes: achievements of ILRI One Health work

The long-term outcomes of the successful implementation of the ILRI One Health work are reduced burden of zoonoses and AMR in people and their livestock, and an increased ability of national governments to anticipate and address high-impact emerging infectious diseases and eliminate their threats, resulting in an overall improvement in global health security [38]. Improved health in livestock will result in increased and more efficient production of livestock-derived foods, benefiting global efforts to enhance food and nutrition security and eradicate poverty.

One Health initiatives led or supported by ILRI have achieved a number of scientific, development, policy, and capacity building outcomes, making progress towards its longer-term outcomes (Table 2). In the area of zoonoses, the Capacitating One Health in Eastern and Southern Africa (COHESA) project for example developed and applied baseline tools, with 11 baseline assessments of national One Health actions completed. On the policy front, COHESA provided technical support for national One Health strategies in multiple countries which could eventually lead to development impacts at the community level. In terms of capacity building, COHESA conducted five workshops focusing on skills such as communication, policy advocacy, project management, and leadership [39].

**Table 2:** Achievements of One Health work.

Dimension	Achievements (program contributions)
One Health cross-cutting work	
Scientific	• Establishment and strengthening of strategic One Health partnerships • Improved understanding of success factors in One Health collaboration • Performed review of One Health in higher education across 15 countries, with subsequent development and implementation of One Health awareness and courses • Performed baseline assessments of national One Health status in 12 countries
Development	• Established multi-stakeholder innovation platforms and One Health units in Ethiopia, Somalia and Kenya, with all service users expressing satisfaction and nearly two-thirds reported accessing services two or more times, primarily human health services followed by animal health [42,43] • Supporting awareness and delivery of rangeland health in Botswana, Zambia and Zimbabwe • Development of tools and approaches for improved AMR (Ethiopia), rabies (Mozambique), and data sharing and solid waste management (Zimbabwe)
Policy	• Establishing and enhancing One Health government platforms in 12 countries with development of OH strategies [39] • Convinced key actors working in One Health (principally biomedical) of the need to include environmental and social science experts • Obtaining new funding from a provincial government in Vietnam to expand the research activities beyond the study sites in the province • Repurposing animal health labs to support human health crises • Contribution to national disease monitoring strategies
Capacity building	• Delivered workshops on communication, policy advocacy, project management, and leadership for 12 national research organizations across 12 countries, strengthening their capacity to drive One Health and improve coordination between research and government sectors within their countries [39] • Organized one regional symposium for policy makers • Built capacity of researchers and implementors to use novel transdisciplinary approaches to better control zoonoses, which was new for many

Zoonoses	
Scientific	• Improved understanding of wildlife farming in Vietnam, bush meat consumption in Côte d'Ivoire, and mixed livestock production in Kenya [37] • Better understanding of human relationships with livestock in the city itself through a social science and economic approach that explores why people keep animals and how they contribute to their livelihoods
Development	• Engaged with government to establish integrated surveillance activities in domestic animals, humans, the environment, and wildlife for the bacterial zoonotic pathogen brucellosis and for *E. coli* as a marker of multi-host transmission of pathogens • Established community surveillance, engaging the community itself to sample the environment and wildlife
Policy	• Government partners allocating staff to support surveillance efforts
Capacity building	• Established the Oloitoktok Zoonoses Research Laboratory, a partnership between the Initiative and the regional government; this site has also been used to extend our pathogen detection work to community pit latrines and wastewater • Established One Health field sites in Thai Nguyen, Vietnam

Food safety	
Scientific	• Conducted Vietnam's first quantitative risk assessment of salmonellosis, a major pork-borne disease [40,41]. • Discovered a larger than expected burden of disease from biological hazards and a smaller burden from chemical hazards • Identified barriers to large-scale adoption of hygienic practices and proposed simpler practices • Better understanding of microbial contamination levels, financial and health burden of foodborne diseases, food safety and nutrition for women and children, health risks of consuming contaminated foods • Estimated the burden of foodborne disease in Ethiopia • Described, measured and modelled major microbial foodborne disease risks in urban systems in Ethiopia and Burkina Faso • Demonstrated that large consumer food safety campaigns can change consumer food safety behaviour in urban Africa
Development	• Informed a World Bank report on food safety, adopted by the Government of Vietnam [40,41]. • Established taskforces for food safety risk assessment, including policymakers and researchers • Joint proposal for a project to support the Kenya Dairy Board to adapt and scale the MoreMilk program into Kenya
Policy	• Integrated the existing technical working group for food safety into the national One Health mechanism in Vietnam • Supported the establishment of a new technical working group for food safety in Ethiopia • African Union-ILRI co-development of government guidelines for engaging the informal food sector towards food safety and food security in Africa
Capacity building	• Developed curriculum benchmarks for One Health and food safety education approved by the Inter-University Council for East Africa • Developed an online food safety and risk assessment course and trained regulators in Ethiopia, Burkina Faso and India • Conducted training of students in risk assessment, risk communication, One Health, system dynamics modeling, and value chain analysis • Incentive-based capacity development program for informal food vendor business to improve food hygiene and handling practices and business skills • Incentive-based capacity development training and coaching program for informal dairy women vendor to improve food hygiene and handling practices and business skills

Antimicrobial resistance (AMR)	
Scientific	• Generated critical scientific data on the drivers of antimicrobial use (AMU) and AMR levels in different livestock systems in different countries in Africa and Asia, integrating a One Health framework for developing context-relevant AMR mitigating solutions [38]. • Created economic models for calculating the cost-benefit of farm-level interventions on reducing the burden of AMR in animals and the broader society • Developed and piloted protocols and tools for semi-intensive farming systems to gather data on disease burden and AMU
Development	• Developed and implemented robust surveillance systems that track AMU and AMR across multiple sectors (animal, human, and environment) using a One Health approach [38].
Policy	• Enhanced the technical and professional capacities of national researchers, animal health professionals, and policymakers through hands-on training and mentorship in AMR diagnostics, surveillance, and governance • Active participation in international advisory boards and Quadripartite technical groups on AMR • Contributed to key national, regional and global policy guidelines
Capacity building	• Mentored and trained government employee working in the human, animal, and aquatic health sectors on AMR diagnostics, AMR and AMU surveillance, and AMR governance • Strengthened national laboratory and surveillance capacities for AMR in Africa and Asia • Developed and disseminated advocacy and communication tools, increasing awareness and understanding of AMR prevention among farming communities and healthcare providers

In the area of food safety, the PigRISK project for instance, conducted Vietnam's first quantitative risk assessment of salmonellosis, uncovering a greater-than-expected burden of disease from biological hazards and a smaller burden from chemical hazards. It also identified barriers to the large-scale adoption of hygienic practices and proposed simpler solutions. On the development side, PigRISK contributed to a World Bank report on food safety that was adopted by the Government of Vietnam and established taskforces for food safety risk assessment, including policymakers and researchers [40,41]. In terms of policy, the establishment of taskforces reflects ongoing efforts to strengthen food safety governance. Capacity-building efforts included training programs for students in risk assessment, risk communication, One Health, system dynamics modeling, and value chain analysis [40].

In terms of AMR, the CGIAR One Health Initiative (OHI) enhanced understanding of livestock feed quality, the drivers of antimicrobial use, and governance related to AMR. Additionally, protocols were developed and piloted to investigate drug quality across different matrices, contributing to improved monitoring and assessment practices. On the development side, efforts are ongoing to design AMR interventions. In terms of policy, OHI contributed to national and regional AMR strategies as well as guidelines for antimicrobial use in livestock. Furthermore, significant capacity-building efforts included training students, with opportunities for overseas placements under the Fleming Fund, and the formation of AMR partnerships with the CGIAR AMR Hub [31], fostering collaboration and knowledge sharing.

It is worth noting the importance of work beyond science. ILRI has focused on capacity development, providing cadres of global experts in One Health across LMICs. The advocacy work drawing on research results is also critical – through research, ILRI has made the world aware of the impact of many One Health threats and how to address them. This has led to governments and donors prioritizing these previously neglected health burdens.

### 2.4 Lessons learned

Over two decades of applying a One Health approach at ILRI, several valuable lessons have emerged that can guide the planning, implementation, and evaluation of One Health agricultural research-for-development efforts:

#### 2.4.1 Planning

• One Health initiatives are inherently complex, involving multiple disciplines and sectors. Institutions should allocate adequate time and financial resources for careful planning and minimize disruptions during implementation. Recognize One Health proposals as valuable outputs in their own right.
• Optimizing health outcomes for people, animals, and the environment simultaneously poses significant practical challenges. Adopting systems thinking can help to identify balanced One Health actions by evaluating the co-benefits and trade-offs of various actions and maximizing overall benefits across domains with minimal adverse effects.
• One Health projects are more effective when they are participatory and driven by the needs of national stakeholders. Design One Health projects to be flexible, participatory, and responsive to emerging needs, allowing national stakeholders to take the lead.
• It is more efficient to build on existing solutions rather than starting from scratch. Identify opportunities to integrate One Health thinking into existing projects.
• Thematic organization of activities by One Health thematic areas promotes disciplinary work but may limit opportunities for synergies, potentially hindering the full potential of the One Health approach. During planning, actively incorporate strategies to foster integration, considering both conceptual and methodological forms, to maximize synergies and enhance the effectiveness of One Health initiatives.
• Interventions should apply a social inclusion approach to address the distinct roles that different groups of people play to address One Health issues. Consider for example gender-responsive interventions that support women and men in their different roles.

#### 2.4.2 Implementation

• Collaboration across diverse, multi-sectoral teams is crucial but can be easily overlooked. Program leads should prioritize ongoing dialogue within and across teams, allocate time and resources for communication, and ensure transparency in staff and partner allocations and work plans to foster a collaborative environment.
• The integration of social sciences into projects can help to enhance understanding of community dynamics and validate qualitative research methods.
• Identifying One Health champions is key to the uptake of the approach. Engaging such champions early in the project can help to ensure successful implementation.
• Higher education institutions are crucial in the One Health institutionalization process, particularly in training current and future One Health professionals. Strengthening the involvement of higher education in One Health by enhancing their capacity to train professionals and integrating One Health concepts into curricula.
• Policy engagement is essential to achieve more substantial impacts. Prioritize policy engagement activities – while ensuring these are ongoing – to ensure that research and interventions influence policy decisions and lead to sustainable outcomes.

#### 2.4.3 Evaluation

• Sustaining effective practices through reflection and documentation has proven valuable. Continue documenting and sharing reflections throughout the program, allocating resources to support these efforts. This practice helps identify areas that are working well and those that need improvement, ensuring ongoing learning and adaptation.
• Sharing experiences across countries at different stages in their One Health pathway can help avoid pitfalls. For projects working in different regions, promote cross-country interactions to facilitate the exchange of knowledge and best practices, enabling countries to learn from one another's experiences.

## 3 Conclusions and future directions

ILRI research has advanced our understanding of challenges and solutions of zoonoses, food safety, and AMR. By challenging conventional wisdom, ILRI revealed that some zoonotic diseases were either more or less common than previously thought, highlighting their impact on human health, wildlife, and ecosystems. In food safety, ILRI's decade-long study of informal markets confirmed that food safety is a growing issue affecting smallholder farmers, with significant implications for human health and livestock production. In AMR research, One Health work played a key role by improving knowledge on antimicrobial use, feed quality, and governance, while also developing drug quality assessment protocols and influencing AMR policies and interventions at national and regional levels.

We recognize some possible limitations of this study. We limited the threshold of USD 1 million budget per project as an inclusion criteria for our review. While this decision helped us to focus on depth of analysis, it may have left out smaller yet important One Health projects at ILRI. ILRI is part of CGIAR and collaborates with other centers. As such, we did examine One Health projects from other CGIAR centers but not systematically. However, we knew that there were very few from other CGIAR centers.

From this experience we can suggest some future directions:

Zoonoses research should focus on establishing systematic data collection and improving our understanding of the spatial distribution of zoonotic diseases to better target control efforts. This requires strengthening collaboration between medical and veterinary sectors through a One Health approach to overcome inter-sectoral barriers. Developing affordable and efficient diagnostics for both human and animal hosts is crucial for managing zoonoses, alongside creating metrics that accurately capture their societal burden, particularly for communities heavily reliant on livestock. Research should also prioritize interventions targeting zoonoses in livestock, as this approach is more cost-effective and prevents human suffering. Additionally, given the ongoing threat of emerging zoonoses, there is an urgent need to enhance surveillance, response strategies, and policy frameworks, while exploring innovative public-private partnerships and market-based solutions to ensure sustainable and scalable control efforts.

Future food safety research should continue to focus on informal markets, where food safety is a growing challenge that impacts human health, livestock production, and market success, particularly for smallholder farmers. ILRI's work has already led to a number of contributions, including innovative tools and strategies that have influenced food safety practices at local, national, and regional levels. As our understanding of FBD in LMICs deepens, future research should aim to gather more evidence on the long-term effects of successful interventions, like the three-legged stool approach for training and empowering market actors, and explore how these approaches can be adapted and applied in different contexts to ensure lasting improvements in food safety. It is recommended to integrate food safety interventions in large programs in nutrition, WASH and empower local authorities to invest in, develop and implement food safety interventions.

Future AMR research should deepen our understanding of the socio-economic and cultural drivers of antimicrobial use, advancing drug quality assessment protocols, and rigorously testing context-specific AMR interventions. Strengthening policy engagement and translating strategies into actionable policies are crucial, alongside expanding capacity-building efforts and integrating AMR education into professional training. Promoting cross-sectoral collaboration through innovative models and leveraging digital platforms will enhance knowledge sharing and global coordination. Finally, incorporating a One Health approach remains essential, particularly in exploring the environmental dimensions of AMR and developing integrated strategies across human, animal, and environmental health.

## CRediT authorship contribution statement

**Hung Nguyen-Viet:** Writing – original draft, Conceptualization. **Steven Lâm:** Writing – original draft, Conceptualization. **Silvia Alonso:** Writing – review & editing. **Fred Unger:** Writing – review & editing. **Arshnee Moodley:** Writing – review & editing. **Bernard Bett:** Writing – review & editing. **Eric M. Fèvre:** Writing – review & editing. **Theodore Knight-Jones:** Writing – review & editing. **Siobhan M. Mor:** Writing – review & editing. **Ha Thi Thanh Nguyen:** Writing – review & editing. **Delia Grace:** Writing – review & editing.

## Declaration of competing interest

No conflict of interest is stated.

## Data Availability

No data was used for the research described in the article.
